# Prevalence of Pertactin-Deficient *Bordetella pertussis* Isolates, Slovenia

**DOI:** 10.3201/eid3011.231393

**Published:** 2024-11

**Authors:** Alex-Mikael Barkoff, Tamara Kastrin, Katja Seme, Marta Grgič Vitek, Jussi Mertsola, Qiushui He

**Affiliations:** National Reference Laboratory for Pertussis and Diphtheria, Institute of Biomedicine, University of Turku, Turku, Finland (A.-M. Barkoff, J. Mertsola, Q. He); InFLAMES Research Flagship Center, University of Turku, Turku (A.-M. Barkoff, J. Mertsola, Q. He); National Laboratory of Health, Environment and Food Department for Public Health Microbiology, Ljubljana, Slovenia (T. Kastrin); Institute of Microbiology and Immunology, Faculty of Medicine, University of Ljubljana, Ljubljana (K. Seme); Communicable Diseases Centre, National Institute of Public Health, Ljubljana (M. Grgič Vitek)

**Keywords:** Bordetella pertussis, bacteria, respiratory infections, acellular pertussis vaccine, pertactin, Slovenia

## Abstract

In Slovenia, primary acellular pertussis vaccines (ACVs) containing pertactin (PRN) were mostly used during 1999–2016; ACVs without PRN were introduced in 2017. Among 123 *Bordetella pertussis* strains collected during 2002–2020, a total of 48 were PRN-deficient; 44 were collected after 2017. Changes to ACVs could increase PRN-deficient *B. pertussis* and infections.

In Slovenia, whole-cell pertussis vaccine was introduced in 1959 and replaced by acellular pertussis vaccine (ACV) in 1999. ACVs containing pertactin (PRN), a highly immunogenic virulence factor of *Bordetella pertussis*, were used during 1999–2016, but since 2017, ACVs with and without PRN have been used, excluding during 2011, when only ACV without PRN was used. During 2006–2016, ACVs with and without PRN were used for primary immunization (Table). Since 2009, a booster vaccine including PRN has been given to children 8 years of age, and coverage for primary and booster vaccinations has been high (>90%). 

**Table:** Pertussis vaccines assessed in a study of prevalence of pertactin-deficient *Bordetella pertussis* isolates, Slovenia*

Year	Primary vaccines with PRN					Primary vaccines without PRN			Booster vaccines with PRN	
	Infanrix†	Zagreb dTP‡	Infanrix/Hib§	Infanrix/IPV+Hib¶		Pentaxim#	Hexacima**		Boostrix††	Adacel‡‡
1999	X	X								
2000			X	X						
2001	X		X							
2002			X							
2003			X	X						
2004				X						
2005				X						
2006				X		X				
2007				X		X				
2008				X		X				
2009				X		X			X	
2010				X		X			X	
2011						X			X	
2012				X		X			X	
2013				X					X	
2014				X					X	
2015				X					X	
2016				X		X			X	
2017						X			X	
2018						X			X	
2019						X			X	
2020						X	X		X	X
2021							X			X
2022							X			X
2023							X			X

*X indicates year vaccine was in use. Vaccination coverage for the primary series has been 90% higher since 2002; booster dose given at 8 years of age was introduced in 2009, and its coverage has ranged from 90.0% to 96.9% since its introduction. DT, diphtheria toxoid; FHA, filamentous hemagglutinin; FIM, fimbriae; HBsAg, hepatitis B virus surface antigen; Hib, *Hemophilus influenzae* type B; IPV, inactivated polio virus; PRN, pertactin; PT, pertussis toxin; TT, tetanus toxoid. †GlaxoSmithKline (https://www.gsk.com); formulation PT-FHA-PRN-DT-TT. ‡Institute of Immunology and Tumor Genetics (https://www.info.hazu.hr); pertussis whole-cell vaccine. §GlaxoSmithKline; formulation PT-FHA-PRN-DT-TT-Hib. ¶GlaxoSmithKline; formulation PT-FHA-PRN-DT-TT-IPV-Hib. #Sanofi Pasteur (https://www.sanofi.us); formulation PT-FHA-DT-TT-IPV-Hib. **Sanofi Pasteur; formulation PT-FHA-DT-TT-IPV-Hib-HBsAg. ††GlaxoSmithKline; formulation PT-FHA-PRN-DT-TT. ‡‡Sanofi Pasteur; formulation PT-FHA-PRN-FIM2-FIM3-DT-TT.

A recent study from Japan showed a decreased frequency of PRN-deficient *B. pertussis* isolates after a change to an ACV without PRN (*1*). Similar observations have been made in Finland (Q. He et al., unpub. data), and partly in Spain (*2*). In Finland, primary vaccination using ACV without PRN was implemented in 2019, and all isolates collected during 2022–2023 were PRN-positive (Q. He et al., unpub. data). Previously, we have shown a high frequency of PRN-deficient *B. pertussis* isolates in Slovenia (*3*); such isolates could evade vaccine-induced immunity. We investigated how the compositional change in the current ACV has affected circulating *B. pertussis* strains.

We studied all 123 *B. pertussis* isolates collected during 2002–2020 in Slovenia, including 27 previously published isolates (*3*). Isolates were collected from different locations; 74 were collected during 2002–2016 and 49 during 2017–2020. Vaccination history was available for 45/49 (91.8%) patients reported during 2017–2020, among whom 31/45 (68.9%) were fully vaccinated. We used a standardized ELISA method with monoclonal antibodies to measure antigen expression of PRN, pertussis toxin (PT), filamentous hemagglutinin (FHA), and fimbriae (Fim) 2 and 3, as described previously (*4*). We used whole-genome sequencing of the *prn* gene to identify mechanisms causing PRN deficiency (*5*). For genotyping, we used PCR-based methods to identify allele specificity of *prn*, *ptxP*, and *ptxA* genes (*6*).

Altogether, 48 (39.0%) isolates did not express PRN. All but 4 isolates collected during 2002–2016 (70/74 [94.6%]) expressed PRN, but 44/49 (91.8%) isolates collected during 2017–2020 did not (Figure). One isolate collected in 2020 did not produce PRN or FHA. All isolates produced PT, and all but 2 isolates produced FHA. Most (43/48 [89.6%]) PRN-deficient isolates carried the IS*481* insertion within the *prn* gene at positions 1613 (n = 40) or 2735 (n = 3). Among the other 5 isolates, 2 had a 22-kb conversion in the promoter region of *prn* gene, 1 isolate had a large deletion of nucleotides within the *prn* promoter–gene area, and 2 others had single nucleotide deletions, 1 at ΔT 632 and 1 at ΔG 793. The mechanism behind FHA deficiencies remains unknown. 

**Figure:**
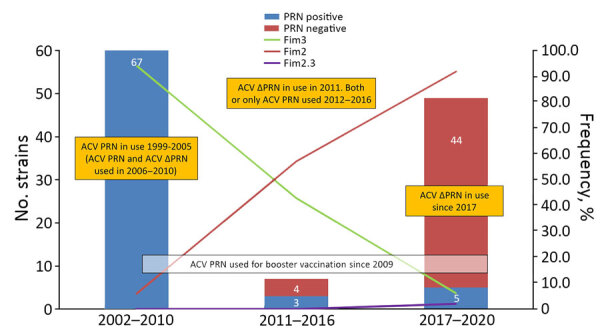
Number of PRN-deficient isolates and frequency of fimbrial serotypes in a study of pertactin-deficient *Bordetella pertussis* isolates, Slovenia, 2002–2020. Vaccines in yellow boxes are those used for primary vaccination; those in the white box are used for booster vaccination. ACV, acellular pertussis vaccine; ACV PRN, ACV containing pertactin; ACV ∆PRN, ACV without pertactin; Fim, fimbriae; PRN, pertactin.

For serotypes, 53/123 (43.1%) isolates harbored Fim2, 69/123 (56.1%) Fim3, and 1/123 (0.8%) Fim2,3. From 2017 onward, 45/49 (91.8%) isolates harbored Fim2, and 66/74 (89.2%) isolates collected during 2002–2016 harbored Fim3 (Figure). Genotyping showed that 122/123 (99.2%) isolates carried *ptxA1*, and 120/123 (97.6%) carried *ptxP3* alleles. For *prn*, *prn2* was dominant 99/123 (80.5%), but *prn*6–8 alleles (23/123, 18.7%) were also notified among the PRN-deficient isolates with an IS*481* insertion. For 1 strain, *prn* was untypable.

Prevalence of PRN-deficient *B. pertussis* isolates remains high in Slovenia, although the current primary vaccines do not contain PRN. That finding differs from findings in Spain, Japan, and Finland (*1*,*2*; Q. He et al., unpub. data), where decline in frequency of PRN-deficient isolates was observed after the change to an ACV without PRN. Although the current vaccination schedule in Slovenia is effective and has high coverage, a 2021 seroprevalence study indicated high circulation of pertussis (*7*), which might affect selection pressure on PRN from natural infection. In addition, since 2009, Slovenia has implemented an additional booster containing PRN in 8-year-old children, which has high vaccination coverage (>90%). That booster could affect the number of PRN-negative strains. In addition, the effect of the frequent changes in vaccines on selection pressure for PRN-deficient isolates cannot be excluded.

We also observed a change in serotype frequency from Fim3 to Fim2 during the study period (Figure). In Slovenia, most ACVs do not contain Fim. Therefore, the change from Fim3 to Fim2 among isolates is likely because of natural selection, which may bias population immunity toward the dominant serotype (*8*). Furthermore, Fim2 strains could express both Fim2 and Fim3 during infection, as described in antibody findings among persons infected by Fim2 *B. pertussis* strains (*9*). Our finding of increasing frequency of both *B. pertussis* PRN-deficiency and Fim2 strains aligns with findings from a study in Spain that suggest a possible link between the 2 characteristics (*2*), which may provide an advantage of the isolates to escape population immunity. Most isolates in this study carried the genotype ptxA1/prn2/ptxP3, which is common in countries using ACVs (*6*).

In conclusion, the unique evading mechanisms of *B. pertussis* against vaccine-induced immunity in Slovenia remain unclear. To maintain optimal vaccination programs to prevent pertussis, Slovenia should continue monitoring circulating *B. pertussis* isolates to inform possible implications for disease incidence and vaccination strategies.
